# Computational selection of antibody-drug conjugate targets for breast cancer

**DOI:** 10.18632/oncotarget.6679

**Published:** 2015-12-19

**Authors:** François Fauteux, Jennifer J. Hill, Maria L. Jaramillo, Youlian Pan, Sieu Phan, Fazel Famili, Maureen O'Connor-McCourt

**Affiliations:** ^1^ Information and Communication Technologies, National Research Council Canada, Ottawa, Ontario, Canada; ^2^ Human Health Therapeutics, National Research Council Canada, Ottawa, Ontario, Canada; ^3^ Human Health Therapeutics, National Research Council Canada, Montreal, Quebec, Canada

**Keywords:** ADC, drug target, microarray, feature selection, ensemble classification

## Abstract

The selection of therapeutic targets is a critical aspect of antibody-drug conjugate research and development. In this study, we applied computational methods to select candidate targets overexpressed in three major breast cancer subtypes as compared with a range of vital organs and tissues. Microarray data corresponding to over 8,000 tissue samples were collected from the public domain. Breast cancer samples were classified into molecular subtypes using an iterative ensemble approach combining six classification algorithms and three feature selection techniques, including a novel kernel density-based method. This feature selection method was used in conjunction with differential expression and subcellular localization information to assemble a primary list of targets. A total of 50 cell membrane targets were identified, including one target for which an antibody-drug conjugate is in clinical use, and six targets for which antibody-drug conjugates are in clinical trials for the treatment of breast cancer and other solid tumors. In addition, 50 extracellular proteins were identified as potential targets for non-internalizing strategies and alternative modalities. Candidate targets linked with the epithelial-to-mesenchymal transition were identified by analyzing differential gene expression in epithelial and mesenchymal tumor-derived cell lines. Overall, these results show that mining human gene expression data has the power to select and prioritize breast cancer antibody-drug conjugate targets, and the potential to lead to new and more effective cancer therapeutics.

## INTRODUCTION

Personalized cancer therapies are expected to be more effective than conventional treatment and to minimize detrimental effects on normal cells [1]. Different strategies have been used in the development of cancer therapeutic monoclonal antibodies (mAbs) including direct and immune-mediated cell killing, and targeting of the tumor neovasculature [2]. To improve clinical efficacy and to overcome some limitations of first generation mAb-based therapeutics, the industry is currently shifting towards innovative and more powerful modalities such as bi-specific antibodies and antibody-drug conjugates (ADCs) [3, 4]. Antibody-drug conjugates offer the ability to deliver potent cytotoxic drugs specifically to tumor cells [5]. Most current ADC development efforts are focusing on cell surface proteins, the binding of which leads to ADC internalization by receptor-mediated endocytosis, and subsequent release of the cytotoxic payload inside tumor cells [6]. The choice of therapeutic target is a critical success factor in this endeavor [7].

The computational selection of therapeutic targets is a multifaceted process that generally starts with mRNA or protein quantitative analysis to identify targets that are overexpressed in tumor cells. Depending on the type of target and the chosen drug and delivery strategy, candidates are then prioritized using various approaches [8–11]. These approaches include filtering based on subcellular localization, molecular interactions and network modeling, analysis of scientific literature and patents, association of expression with survival, genotype-phenotype analysis, and integration of knowledge from drug and clinical databases. The main criterion for ADC target selection is tumor specificity, to avoid toxicity in vital organs and tissues [7, 12]. To evaluate this property using gene expression data, the analysis of “as many samples as possible” is necessary given the naturally important phenotypic variation within and between individuals [13] and additional noise resulting from sample handling and other experimental procedures [14]. The Gene Expression Omnibus (GEO) [15] is a major functional genomics data repository, currently offering public access to over 100,000 samples analyzed with the Affymetrix Human Genome U133 Plus 2.0 GeneChip [16]. Compiling homogeneous, reliable information using variably granular, semi-structured sample annotations is difficult, while critical for the overall quality of findings in studies reusing public gene expression data [17]. A GEO metadata SQL database was created in 2008 [18], which made records amenable to programmatic analysis. Although sample annotations provide a basis for analysis, various technical problems such as inaccuracy in receptor testing [19] or a low percentage of tumor cells may affect the relevance of a given sample to a given class. Classification techniques [20] are thus essential to validate and refine annotation-based class labels.

Feature selection is another critical aspect of computational target selection. In a recent review, Saeys *et al.* [21] divided bioinformatics feature selection techniques into three categories depending on if and how the feature search is combined with the classification model. The most common approach to select features in microarray data consists in ranking and filtering features using the Student *t*-test [22] or the analysis of variance (ANOVA) *F*-test [23]. Limma, a popular software package used for differential expression analysis, fits a linear model to expression data for each gene, and variance estimates are adjusted by borrowing information across genes [24, 25]. Problems associated with parametric methods include distributional assumptions and the dependence of *p*-values on sample size [26, 27]. Another class of supervised feature selection techniques makes use of weights acquired in the construction of classifiers such as random forests [28] or support vector machines [29]. These methods, though very effective, do not yield representations that are directly interpretable. In this paper, we used the coefficient of overlap of kernel densities, a concept previously used in social statistics [30], adapted here using a locally adaptive form of the kernel density estimate [31, 32], as a bioinformatics feature selection algorithm. The algorithm is easy to interpret, does not depend on sample size, accommodates various distributions, and is shown to perform equally or better than the above methods in breast cancer and tumor-derived cell line classification problems.

In this study, we have applied feature selection and classification methods to identify candidate therapeutic targets in breast cancer, the most common cancer in women and a heterogeneous disease in nature [33]. Breast cancer is categorized in three basic therapeutic groups associated with distinct molecular subtypes, based on the status of the estrogen receptor (ER), the progesterone receptor (PR), and the receptor tyrosine-protein kinase erbB-2 (ERBB2, *a.k.a.* HER2) [34]. Although further subdivisions could have been made in each group, we focused our analysis on the molecular subtypes associated with these three basic therapeutic groups (luminal, HER2+ and triple-negative). Over 4,500 breast cancer samples were collected and classified into these three molecular subtypes. For the selection of candidate ADC targets overexpressed in each breast cancer subtype, differential gene expression analysis was performed against over 3,500 samples from a range of vital organs and tissues. Although ADC strategies generally rely on their internalization by cancer cells, a recent report [35] suggests that non-internalizing ADCs targeting the tumor microenvironment may also be effective. For this reason, and also to provide candidate targets for alternative modalities such as antibody-radionuclide conjugates [36], we included both cell surface and extracellular proteins in the analysis. We also aimed to prioritize targets linked with metastasis, since this is the main cause of mortality in patients with solid tumors including breast cancer [37]. Metastasis involves a series of steps where specific tumor cells break through the basement membrane and invade subjacent stromal cell layers, and traverse the endothelium into blood microvessels where they travel to and infiltrate distant sites [38]. The first step in this series of events involves phenotypic changes in subpopulations of cells at the invasive margins of carcinomas, which acquire traits that are important for motility and dissemination, a conversion called the epithelial-to-mesenchymal transition (EMT) [39]. Resistance to therapy and recurrence have been linked with stem cell properties of mesenchymal cells including self-renewal, motility, resistance to apoptosis, cell cycle arrest, suppression of immune responses and enhanced drug transport [40, 41]. Many of the phenomena surrounding EMT and metastasis have been studied in cell line models [42, 43]. Here, we performed classification and differential gene expression analysis in a large collection of tumor-derived cell lines [44, 45], to further prioritize targets linked with the mesenchymal phenotype and metastasis.

## RESULTS

**Figure 1:**
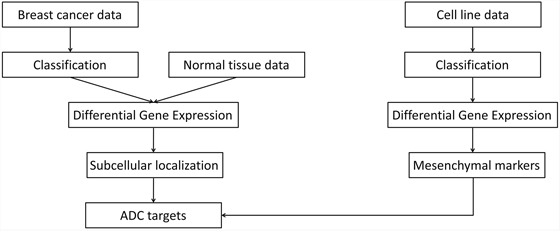
Overview of the approach for target selection and prioritization. ADC, antibody-drug conjugate

Our approach for target selection and prioritization is schematized in Figure 1. In brief, breast cancer samples were classified into three molecular subtypes. Differential gene expression analysis was performed against normal tissues to identify genes overexpressed in each subtype. Subcellular localization information was used in conjunction with gene expression data to select a primary list of cell surface and extracellular candidate targets. In parallel, differential gene expression analysis was performed in epithelial against mesenchymal tumor-derived cell lines to identify, among selected targets, those also potentially linked with EMT.

### Breast sample classification

**Figure 2:**
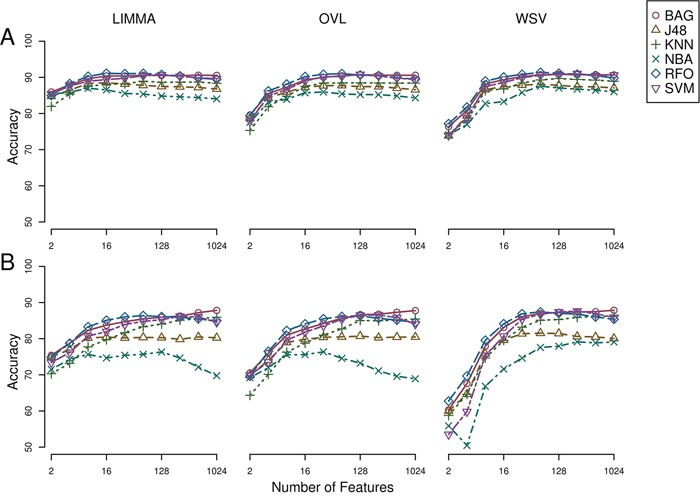
Repeated cross-validation of class labels assigned using receptor status in breast cancer and normal breast tissue samples A) One-against-one feature selection and classification. B) One-against-all feature selection and classification. LIMMA, linear models for microarray; OVL, overlap of locally adaptive kernel densities; WSV, weight of support vectors; BAG, bagging; J48, C4.5 decision tree; KNN, k-nearest neighbors, NBA, naive Bayes; RFO, random forests; SVM, support vector machine.

**Figure 3:**
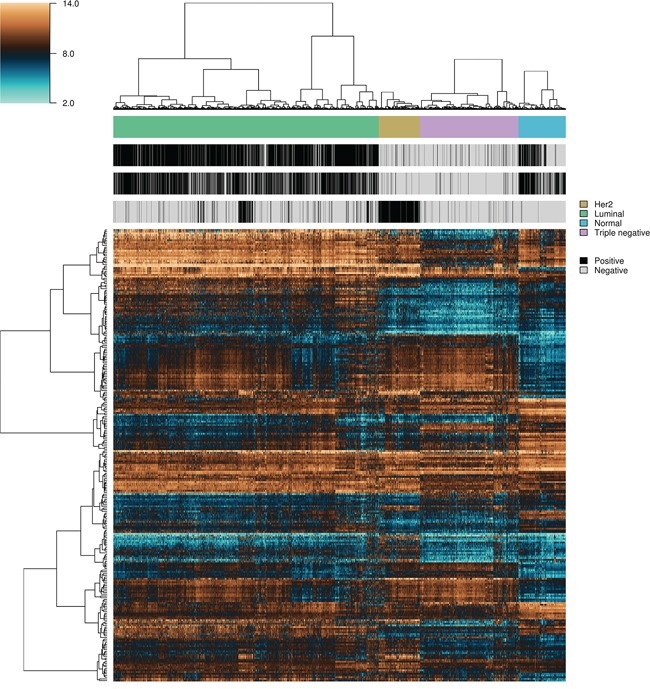
Heatmap and intra-class hierarchical clustering of 5,259 breast cancer and normal breast tissue samples Top and lower horizontal color bars show sample class and receptor status (ER, PR and HER2), respectively. The top gene dendrogram represents features used for the binary classification problems involving breast cancer against normal samples, and the lower gene dendrogram comprises features used for binary classification problems involving the different breast cancer molecular subtypes.

Breast samples (total of 5,379) were initially assigned to one of four classes: normal, luminal, HER2+ and triple-negative, based on sample annotations and receptor status. Class labels were validated using repeated cross-validation combining three feature selection methods, six classification algorithms and two multiclass classification strategies (Figure 2). The performance of all approaches was compared using analysis of variance. The kernel-based feature selection technique slightly surpassed the other two algorithms (p<1E-3). The other factors (multiclass classification strategy, classification algorithm and number of features) all affected performance (p<1E-10). The accuracy under one-against-one (OAO) classification was higher than under one-against-all (OAA) classification. The best performing classification algorithms were: support vector machines (SVM), random forests (RFO) and bagging, followed by *k*-nearest neighbors (KNN), J48 and naïve Bayes. Accuracy increased as the number of features increased in ${\left(2^{k}\right)}_{k=1}^{5}$ and remained relatively stable in ${\left(2^{k}\right)}_{k=5}^{10}$. Classification accuracy was close to 90% for two of the best performing classification algorithms (SVM and RFO) in combination with the three feature selection methods, with feature numbers in ${\left(2^{k}\right)}_{k=5}^{10}$, under OAO classification. This information was used in model selection for iterative ensemble classification. For the final classification of breast samples, filter-based feature selection was performed by selecting the top ${\left(2^{k}\right)}_{k=5}^{10}$ ranking features using three statistics (*q*-value from Limma, overlapping coefficient of kernel densities and weight of SVM), and at each iteration classifiers were trained on all labeled instances. In total, 36 predictions were made for each sample (three feature selection methods, two classification methods, and six increasing number of features). Labels assigned with high confidence (>95% of votes) by the ensemble of experts were fed back into the data and used for subsequent feature selection and training of the classifiers. Complete convergence was achieved after 15 iterations. At this point, 5,107 samples were assigned class labels unanimously among experts (100% of votes), and 70 samples were assigned class labels with high confidence (>95% of votes). An additional 82 samples were labeled with reasonable confidence (>75% of votes), and 120 samples were left unlabeled. As a final result, a total of 5,259 samples were labeled, in the following classes: 549 normal, 3,085 luminal, 479 HER2+ and 1,146 triple-negative breast cancer. Among labeled samples, 4,808 (91%) retained original labels assigned using sample annotations and receptor status. Figure 3 shows representative gene expression of markers used for classification in the above classes. The top 64 genes for each of six binary classification problems were selected and hierarchical clustering was performed within each class, and within two groups of genes, the first one consisting of features used for the binary classification problems involving breast cancer against normal samples, (Figure 3, top gene dendrogram), and the second group comprising genes used for classification problems involving breast cancer molecular subtypes (Figure 3, lower gene dendrogram). Distinct gene clusters with low and high expression characteristic of each class are clearly visible.

### Cell line classification

**Figure 4:**
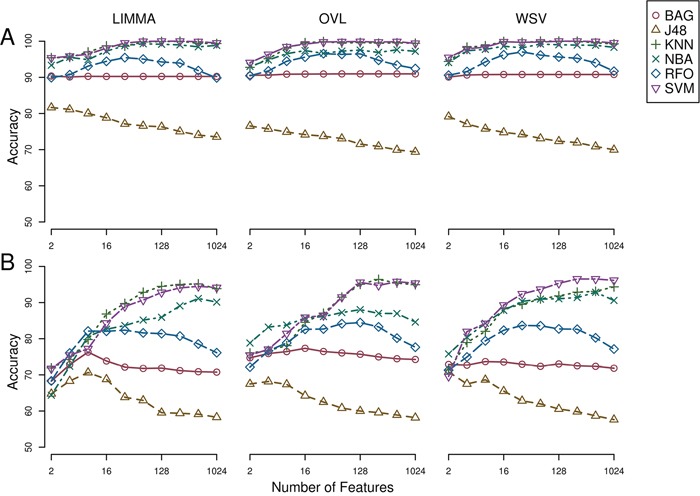
Repeated cross-validation of assigned class labels in NCI60 cell lines A) One-against-one feature selection and classification. B) One-against-all feature selection and classification. LIMMA, linear models for microarray; OVL, overlap of locally adaptive kernel densities; WSV, weight of support vectors; BAG, bagging; J48, C4.5 decision tree; KNN, k-nearest neighbors, NBA, naive Bayes; RFO, random forests; SVM, support vector machine.

**Figure 5:**
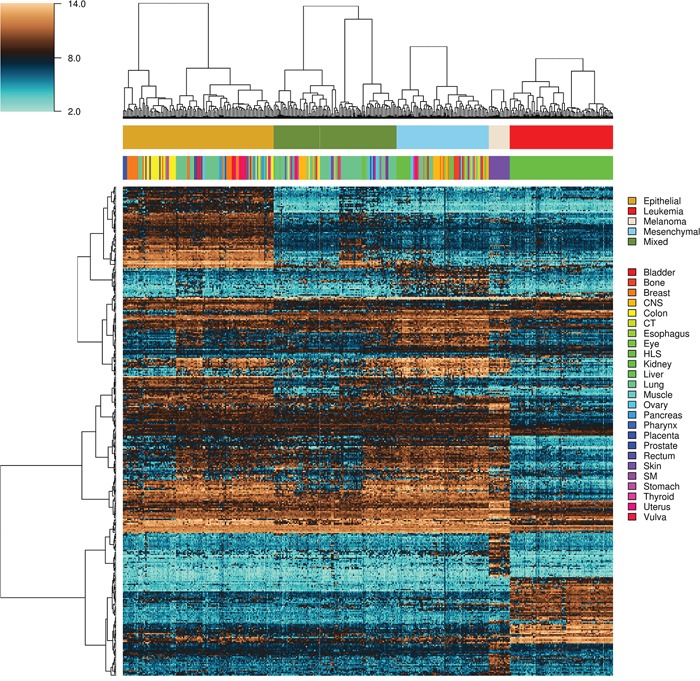
Heatmap and intra-class hierarchical clustering of 351 tumor-derived cell lines Top and lower horizontal color bars show cell line class and tissue origin, respectively. The top gene dendrogram represents features used for the binary classification problems involving the epithelial, mixed and mesenchymal classes only, and the lower gene comprises genes used for binary classification problems involving the melanoma and the leukemia classes. CNS, central nervous system; CT, connective tissue; HLS, hematopoietic and lymphatic system; SM, synovial membrane.

**Figure 6:**
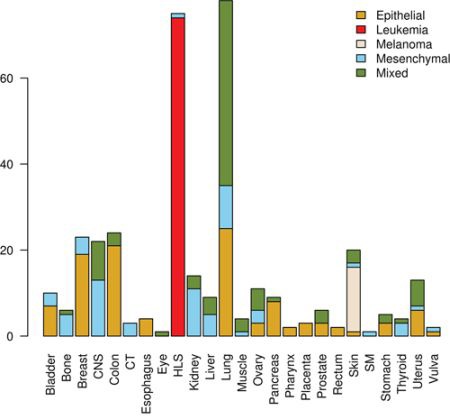
Distribution of tissues of origin and classes in tumor-derived cell lines CNS, central nervous system; CT, connective tissue; HLS, hematopoietic and lymphatic system; SM, synovial membrane.

Epithelial and mesenchymal cell lines were identified in a collection of 359 cell lines, to perform differential expression analysis and identify candidate targets linked with EMT and metastasis. We used results from a previous study in the NCI60 panel [46] to identify five groups of cell lines having homogeneous gene expression patterns in respect to four major gene clusters. These groups of cell lines were assigned the following class labels: epithelial (10), mesenchymal (23), mixed (12), melanoma (8) and leukemia (6) (Supplementary Data, Table S1). Class labels were validated using repeated cross-validation (Figure 4). The accuracy for each combination of factors was similar to that obtained in the classification of breast samples, although in this case the three feature selection methods performed equally well, and the best performing classification algorithms were SVM and KNN, followed by naïve Bayes, RFO, bagging and J48. Classification accuracy was close to 100% for the two best performing classification algorithms in combination with the three feature selection methods, with feature numbers in ${\left(2^{k}\right)}_{k=5}^{10}$, under OAO classification. For the iterative classification of 300 additional cell lines, feature selection was performed independently in three sets of NCI60 replicates, classifiers were trained on each set of replicates, and predictions were made on each unlabeled cell line replicate. In total, 324 predictions were made for each cell line (three sets of labeled replicates, three unlabeled replicates, three feature selection methods, two classification methods, and six increasing number of features). In this case, convergence was achieved after seven iterations. At this point, 266 of the initially unlabeled cell lines were assigned class labels unanimously among experts (100% of votes), and six cell lines were assigned class labels with high confidence (>95% of votes). An additional 20 cell lines were labeled with reasonable confidence (>75% of votes), and the remaining 8 cell lines were left unlabeled. Of the total 359 cell lines, 108 were labeled as epithelial, 88 as mixed, 66 as mesenchymal, 15 as melanoma and 74 as leukemia (Supplementary Data, Table S2). Representative gene expression of markers used for classification in the above cell lines classes is shown in Figure 5. The top 64 genes for each of ten binary classification problems were selected, hierarchical clustering was performed within each class, and within two groups of genes, the first one consisting of features used for the binary classification problems involving the epithelial, mixed and mesenchymal classes only (Figure 5, top gene dendrogram), and the second group comprising genes used for classification problems involving the melanoma and the leukemia classes (Figure 5, lower gene dendrogram). Gene expression was highly homogeneous and characterized by major gene clusters with low and high expression within each class. Two classes (leukemia and melanoma) were dominated by specific tissues and the other classes (epithelial, mesenchymal and mixed) contained cell lines from many different tissues (Figure 6).

### Target selection and prioritization

Among 19,674 probesets defined in the Entrez Gene custom CDF [47], 18,282 were matched to human genes from the HUGO Gene Nomenclature Committee (HGNC) database of human genes [48], and 16,811 were of type “protein-coding gene”. From this list of genes, a total of 1,713 genes respected the following conditions and were included in a list of potential cell membrane targets: gene ontology annotation [49] contained GO:0005886 (plasma membrane) or Uniprot subcellular location annotation [50] contained the following term: “cell membrane”, and Uniprot topological domain annotation contained at least one transmembrane and one extracellular domain. In addition, 1,369 genes respected the following conditions and were included in a list of potential extracellular targets: gene ontology annotation contained GO:0005576 (extracellular region) or Uniprot subcellular location annotation contained one of the following terms: “extracellular matrix”, “extracellular space” or “secreted”. To identify genes overexpressed in cancer, gene expression in breast cancer samples was compared with gene expression in normal tissues. For each breast cancer molecular subtype (luminal, HER2+ and triple-negative), genes were ordered according to the mean rank of differential expression in cancer versus normal tissues, and the top 50 membrane and extracellular protein-coding genes were retained (Tables 1 and 2). Batch effects were evaluated using series (experiments) as a surrogate, and were found to be random and relatively inert as compared with the tissue and status effects (Supplementary Data, Figures S1 and S2). Epithelial and mesenchymal marker genes were identified by comparing gene expression in corresponding cell line classes, and filtering genes with a differential expression ratio greater than 2 or smaller than 0.5, and an overlap of kernel densities smaller than 0.6. Four membrane and 13 extracellular targets were also in the mesenchymal gene set (Tables 1 and 2).

**Table 1:** Membrane ADC targets, mean expression in breast cancer and normal tissues

	Breast cancer			Normal tissues										
	HER2	LUM	TN	Blood	BM	Breast	Colon	Heart	Kidney	Liver	Lung	Pancreas	Skin	Stomach
Mesenchymal targets														
ADAM12	9.48	9.61	9.09	5.99	4.87	8.58	5.19	5.99	6.03	5.29	6.14	7.02	6.27	6.41
CDH11	9.96	10.16	9.52	4.37	4.88	9.19	7.64	8.34	8.40	6.10	10.08	8.55	8.63	9.45
F2RL2	7.57	8.06	6.88	4.88	4.23	7.08	5.34	6.11	5.11	6.56	4.97	6.92	6.43	7.55
FAP	10.07	10.09	9.53	3.97	3.30	8.58	3.93	5.65	4.71	4.47	7.32	8.09	8.04	5.64
Epithelial targets														
CDH1	10.82	10.41	9.92	4.81	7.44	9.44	11.19	4.07	9.40	10.07	9.46	10.12	10.25	9.29
CDH3	9.92	8.40	10.28	3.61	3.49	9.52	4.40	4.20	7.54	3.96	6.86	5.93	9.61	5.13
EPHB3	8.56	8.51	9.26	6.02	6.10	8.67	8.21	6.57	6.99	6.51	7.29	8.12	8.90	9.70
ERBB2	12.84	9.57	8.51	6.15	5.48	8.20	8.71	8.74	8.90	7.63	8.36	7.69	8.67	8.56
ERBB3	10.78	11.37	10.15	5.53	5.27	10.08	11.47	7.96	10.50	10.58	10.06	9.60	10.52	10.64
IGSF9	9.30	8.47	8.63	4.54	4.52	6.32	9.17	5.65	5.16	7.19	4.75	5.59	8.07	6.83
ITGB6	9.88	8.22	7.92	3.30	3.35	7.86	8.16	5.67	8.22	4.24	9.15	7.62	6.40	6.74
MUC1	11.02	11.86	9.87	5.80	7.29	9.62	10.85	6.32	10.66	5.71	12.04	10.52	8.38	12.19
MUC16	4.48	3.94	6.76	2.62	2.77	4.48	2.59	3.95	2.50	2.54	3.89	2.81	3.56	4.23
PVRL4	9.23	8.49	8.73	5.59	5.96	7.48	6.09	6.48	6.49	5.50	6.65	6.58	8.88	6.82
TLCD1	9.11	8.36	8.21	4.66	5.47	6.56	8.26	6.70	8.10	7.44	7.52	6.99	8.80	7.03
Other targets														
BAMBI	10.88	10.56	10.77	6.67	8.07	10.18	8.89	10.02	10.24	9.72	9.58	8.95	8.35	8.34
BMPR1B	6.69	9.32	6.89	4.29	4.82	6.73	5.19	7.44	8.37	6.31	7.11	7.31	6.55	8.25
CA12	9.30	11.86	8.27	5.74	5.77	9.86	12.59	6.95	12.82	6.08	7.81	9.73	11.53	8.93
CLSTN2	6.11	8.84	5.62	3.67	4.67	8.13	4.70	7.43	7.08	5.32	5.44	6.16	6.35	6.98
CT83	5.21	4.40	7.66	4.31	4.37	4.60	4.32	5.42	4.57	4.33	4.34	4.83	4.16	5.49
FPR3	8.92	8.37	8.93	6.01	5.43	7.51	7.50	7.57	6.76	7.88	8.56	7.90	7.23	7.53
FZD7	9.05	9.41	10.48	4.68	5.86	11.37	8.39	9.40	8.93	6.58	9.10	8.48	10.21	9.76
GABRP	7.64	7.39	11.53	3.26	3.08	11.48	4.27	5.29	6.34	6.07	4.79	6.06	8.21	6.96
GPNMB	13.19	12.82	13.15	6.09	7.79	13.19	10.74	13.08	10.80	10.44	12.57	11.67	13.92	11.59
GPR19	6.32	5.57	7.08	6.94	5.50	4.78	4.65	4.83	4.14	4.52	4.26	6.08	4.41	5.60
GRIA2	3.10	5.71	3.66	2.07	2.47	5.21	2.17	2.94	3.47	3.04	2.39	5.36	4.91	5.66
KCNS3	9.85	9.68	9.12	3.91	5.08	9.37	8.63	7.91	8.11	7.77	10.47	8.80	8.89	9.38
KIAA1324	10.06	10.67	8.48	8.79	5.85	10.00	9.46	5.68	4.62	5.11	7.65	10.56	7.74	10.03
LAMP5	7.98	8.69	7.10	6.99	6.96	6.23	5.08	5.33	5.87	5.62	6.03	7.27	5.66	6.36
LDLRAD3	10.07	10.70	10.46	8.71	7.71	10.49	7.06	8.07	7.77	7.80	8.20	9.13	10.75	8.77
LRP8	8.30	7.27	8.67	7.47	7.90	6.42	6.36	7.00	6.34	5.58	7.14	6.90	6.96	7.25
MMP14	8.88	8.74	8.69	5.47	5.43	7.17	6.74	7.39	7.27	6.33	7.50	7.46	7.85	8.45
NKAIN1	6.02	7.95	5.88	4.30	4.67	5.31	4.95	5.17	4.96	5.43	4.79	5.86	6.04	6.59
NPY1R	6.76	10.95	8.55	3.26	4.36	11.73	9.39	7.16	11.49	10.01	8.11	10.25	10.98	8.36
PMEPA1	10.88	10.43	10.45	6.80	5.98	10.24	8.87	9.58	8.81	7.12	9.46	9.98	9.35	9.29
PRLR	8.30	8.61	7.42	5.15	5.65	7.19	6.61	6.31	7.77	7.21	5.37	6.85	6.12	7.17
PROM1	10.01	7.94	11.23	4.88	8.04	10.08	11.50	8.46	10.71	7.33	7.89	9.85	7.61	10.59
PTPRT	5.13	7.53	4.78	4.43	4.40	6.72	4.94	5.83	5.51	5.21	5.51	6.10	4.99	6.20
SDC1	12.12	11.00	11.26	6.02	7.10	9.51	10.82	5.30	11.26	12.33	10.88	9.36	12.43	9.96
SLC16A6	8.60	9.74	7.56	9.90	8.65	7.51	6.15	6.70	6.65	7.04	8.22	7.27	8.54	7.48
SLC2A10	11.00	11.01	9.30	2.71	5.83	10.09	9.78	7.05	7.78	10.88	9.40	9.72	8.71	9.59
SLC39A6	10.16	12.20	9.96	8.24	8.58	10.73	8.08	8.89	9.49	8.26	9.12	9.76	10.00	9.19
SLC4A11	8.66	7.42	8.04	5.87	5.74	7.48	5.96	7.00	8.37	6.09	6.52	6.66	7.86	7.49
TPBG	11.59	12.31	11.17	6.56	7.08	11.44	10.21	9.45	9.59	7.91	9.84	10.21	10.90	10.71
TREM2	8.37	8.53	8.12	4.32	4.20	7.06	5.47	6.29	4.39	4.62	8.36	6.13	4.98	5.49
TRPV6	8.68	6.09	7.25	5.07	4.68	6.20	4.70	5.76	6.30	6.00	4.20	7.94	6.92	7.50
TTYH1	4.77	4.29	6.30	3.59	3.80	5.87	3.78	4.99	3.54	4.55	3.00	5.70	4.70	4.94
UNC5A	6.50	5.81	4.28	3.67	3.64	4.41	2.97	4.75	3.41	3.55	3.54	4.34	2.70	5.45
VANGL2	8.31	8.36	9.37	5.39	5.58	8.24	7.40	7.58	7.72	6.58	7.70	7.72	9.76	8.73
VTCN1	8.92	9.44	10.10	4.19	4.64	10.30	4.12	5.52	8.47	5.76	5.19	8.35	7.00	6.34

HER2, HER2-positive; LUM, luminal; TN, triple-negative; BM, bone marrow.

**Table 2:** Extracellular ADC targets, mean expression in breast cancer and normal tissues

	Breast cancer			Normal tissues										
	HER2	LUM	TN	Blood	BM	Breast	Colon	Heart	Kidney	Liver	Lung	Pancreas	Skin	Stomach
Mesenchymal targets														
COL11A1	10.88	10.64	10.42	3.21	3.75	5.86	3.62	3.50	4.24	3.79	4.35	6.02	7.10	4.19
COL12A1	10.24	10.27	9.53	3.75	4.43	8.41	6.28	8.10	7.37	6.23	7.79	7.95	9.22	8.49
COL1A1	14.13	14.02	13.23	4.70	7.43	11.35	9.49	10.56	9.22	9.43	10.67	11.45	12.90	12.30
COL1A2	14.62	14.72	14.20	5.35	7.12	13.50	10.95	11.78	10.46	10.06	12.61	12.69	13.79	12.90
COL3A1	14.49	14.60	14.17	3.82	5.87	13.80	12.26	12.20	11.25	11.81	12.51	13.08	13.73	13.73
COL5A1	11.45	11.30	10.64	4.47	5.76	9.70	8.25	9.16	7.51	8.12	9.30	9.25	10.43	10.64
COL5A2	12.45	12.36	11.79	4.84	5.16	10.95	8.75	9.59	8.39	8.97	9.95	10.21	10.87	10.86
COL6A3	13.84	14.00	13.31	6.30	6.87	13.67	10.57	12.48	9.84	10.28	12.86	12.60	12.99	13.05
COL8A1	9.13	9.28	8.64	4.23	4.51	7.98	5.37	8.18	6.95	5.73	9.08	7.42	6.69	7.45
FN1	12.77	12.56	12.03	4.64	4.97	9.64	8.50	10.24	8.54	12.59	12.24	9.51	9.79	10.30
INHBA	9.58	9.44	9.35	3.71	8.08	6.07	3.51	6.54	5.12	7.59	8.45	7.13	6.21	5.65
POSTN	13.62	13.89	13.19	3.63	4.43	12.11	10.54	10.00	9.43	8.16	11.25	9.71	12.79	12.30
THBS2	12.83	12.69	12.18	5.69	5.51	12.04	6.24	10.50	9.03	9.83	9.59	10.62	12.64	9.76
Epithelial targets														
AZGP1	12.94	13.21	11.30	3.78	4.29	13.16	6.83	10.05	11.65	13.70	8.30	11.15	12.58	10.21
Other targets														
AEBP1	12.54	12.35	11.43	6.94	7.42	10.92	7.88	9.01	9.56	9.09	10.53	9.53	11.08	10.37
AGR3	7.34	12.61	5.33	2.93	3.48	8.98	13.24	3.66	5.31	3.27	12.27	9.55	6.70	8.04
ASPN	10.83	11.50	10.08	4.50	4.40	10.01	7.68	10.56	8.87	10.00	9.64	10.44	9.68	10.32
BRINP3	6.87	3.53	3.93	2.73	2.68	3.28	7.45	4.48	5.10	2.80	5.18	4.38	3.14	3.67
CHI3L1	10.08	9.34	11.62	11.32	12.17	9.56	5.08	6.33	9.29	10.02	9.26	7.51	11.12	6.59
CILP	10.66	11.34	9.70	6.38	6.03	11.10	7.36	10.11	7.52	7.58	7.62	9.18	10.87	9.55
COL10A1	11.07	11.20	10.07	5.97	6.17	6.27	5.31	5.88	5.93	6.41	6.54	8.05	5.89	6.43
COMP	9.79	9.88	8.63	4.41	3.88	5.72	3.83	5.49	4.73	3.86	5.81	6.26	8.63	5.21
CST1	7.39	7.32	6.40	3.60	3.55	3.55	3.75	4.09	3.08	3.61	3.61	4.58	4.32	3.89
CTHRC1	13.16	13.07	13.08	4.20	6.55	11.38	7.23	8.30	7.44	6.27	9.32	10.62	11.55	8.99
CXCL10	11.36	10.12	11.78	7.11	6.55	7.98	8.04	8.00	8.31	9.89	9.49	8.45	7.15	8.77
CXCL11	9.11	7.87	9.27	3.91	4.16	5.54	5.71	4.94	5.48	6.22	7.38	8.09	5.11	6.43
CXCL13	9.53	8.19	9.71	3.20	3.35	4.86	8.48	4.01	3.56	5.90	6.00	5.79	3.85	8.85
CXCL9	11.39	10.06	11.32	6.16	6.06	8.64	8.14	7.90	8.15	9.88	9.57	8.56	7.75	8.45
EPYC	6.77	6.18	6.09	2.66	3.52	4.00	3.03	4.56	3.60	3.03	3.31	4.77	2.89	4.51
FDCSP	6.98	6.60	10.32	3.55	3.62	8.41	7.91	4.03	3.45	4.48	4.60	6.05	4.52	7.47
GRP	7.84	8.71	6.83	3.99	3.75	7.56	4.12	4.72	4.48	4.73	7.45	6.24	5.21	8.28
IBSP	6.99	6.14	6.51	4.08	4.28	3.24	3.44	5.31	3.87	4.55	3.30	4.38	3.47	5.28
IL4I1	7.73	6.96	8.15	5.74	4.67	4.95	5.40	5.64	5.07	5.53	5.76	6.08	5.69	6.03
LUM	13.80	13.80	13.18	2.68	5.20	13.63	11.24	12.48	12.00	10.27	13.66	13.24	12.28	12.88
MATN3	6.57	7.82	6.11	2.55	2.86	5.58	3.58	3.10	5.32	2.94	7.43	5.53	5.45	3.80
MDK	10.07	9.55	9.43	4.86	5.96	8.09	8.14	6.22	6.60	6.17	7.93	8.07	6.32	8.20
MFAP2	9.47	9.69	10.09	2.32	3.64	7.71	4.64	5.35	4.98	4.77	9.13	8.58	8.99	7.80
MGP	13.36	14.17	13.82	4.21	4.95	14.48	8.78	13.63	13.00	8.76	13.64	13.16	12.22	13.34
MMP1	9.46	7.05	9.60	4.14	3.30	3.49	7.23	4.37	5.63	3.50	6.09	8.79	4.57	8.50
MMP11	9.60	9.49	8.85	5.10	5.33	6.00	5.93	7.55	6.61	5.37	5.46	7.09	5.67	7.84
MMP12	8.24	7.10	9.63	4.31	4.98	6.60	10.16	6.46	6.35	4.99	6.62	7.94	6.27	7.96
MMP13	8.32	7.67	7.48	3.74	4.41	4.01	3.47	4.38	3.65	4.39	3.70	4.47	3.77	4.82
MMP3	9.01	8.72	8.82	4.64	4.89	7.30	6.47	6.05	5.01	4.95	4.47	6.13	6.06	7.91
MMP7	9.04	9.02	11.65	4.09	4.86	10.94	5.84	6.79	11.22	7.51	9.23	11.38	9.29	9.07
MUCL1	13.93	10.70	8.81	3.84	4.48	13.03	4.57	5.79	5.07	4.67	5.25	5.35	13.44	6.28
MXRA5	12.84	12.69	12.28	4.13	5.90	11.95	9.47	9.94	9.54	9.41	9.68	10.53	12.17	11.36
SCUBE2	8.39	11.43	7.35	4.47	3.90	10.78	8.03	6.98	5.44	5.97	7.77	8.41	9.14	9.07
SFRP4	9.63	10.06	9.14	4.87	5.38	10.51	5.37	7.66	6.96	5.69	8.34	9.12	7.13	8.30
STC2	7.87	10.45	8.27	4.36	4.86	10.36	4.75	7.48	7.17	5.14	7.12	8.45	6.33	6.66
ZG16B	9.87	10.73	8.26	7.03	7.88	9.00	9.62	4.58	3.81	4.87	5.75	6.31	9.87	9.21

HER2, HER2-positive; LUM, luminal; TN, triple-negative; BM, bone marrow.

## DISCUSSION

“Ideal” targets, with very high expression in one or more tumor types and very low expression in all normal tissues are rare. The best example of such targets is ERBB2, which is incidentally the only target for which an ADC (trastuzumab emtansine) is currently commercialized for the treatment of breast cancer [51]. In this study, to select candidate ADC targets, we performed gene expression analysis in three breast cancer subtypes versus a range of normal organs and tissues. Our results show that metadata mining and sample classification are instrumental in the assembly of large datasets representative of patient populations, and that feature selection methods and the incorporation of biological knowledge are essential for the selection of clinically relevant targets. Although currently available ADC target data may be too scarce for a formal discussion about sensitivity and specificity, the selection of targets for which ADCs are in clinical development is nevertheless a good indicator of the validity of our approach.

Our list of cell membrane candidates contained one target for which an ADC is already in clinical use and six additional targets for which antibody-drug conjugates are in clinical trials for the treatment of breast cancer and other solid tumors (Table 3). Combining a recent review on ADCs in clinical trials [52] and a search of the ClinicalTrials.gov database [53, 54] revealed that our list of cell membrane targets contained the majority of target antigens for ADCs in clinical development for the treatment of breast cancer, with the possible exception of the tumor-associated calcium signal transducer 2 (TACSTD2), a recently identified ADC target for triple-negative breast cancer [55]. In our analysis, we found that this target, although displaying a high level of expression in breast cancer, also had a relatively high expression in a number of normal organs and tissues including the skin, lungs, and kidneys and did not score high for this reason. According to the list presented in [6], the only candidate target in our list for which an ADC was previously discontinued is mucin-1 (MUC1), for lack of efficacy in ovarian cancer therapy [56, 57]. Clinical efficacy, however, does not depend only on the selected target but also on the design and components of the ADC (drug, linker and antibody). In fact, there is still interest in MUC1: an ADC targeting a specific glycol-epitope of MUC1 (SAR-566658), as well as an anti-MUC1 chimeric antigen receptor (CAR) T cell therapy are currently in clinical trials [54]. Other selected targets in clinical trials for CAR T cell therapy, an approach that also requires highly tumor-specific targets [58–60], include ERBB2, mucin-16 (MUC16), prominin 1 (PROM1) and the prolyl endopeptidase FAP (FAP) [54]. Overall, the high proportion of clinically relevant targets suggests that our target selection method is valid and that our list of targets may contain new candidates with a high potential for ADC development. A number of these are at various stages of pre-clinical research and development, which further validates our findings. Selected examples are discussed below, with a focus on triple-negative breast cancer.

**Table 3:** Selected targets for ADCs in clinical trials for the treatment of breast cancer and other solid tumors

Target	Drug	Company
ERBB2	T-DM1	Roche/Genentech
GPNMB	CDX-011	Celldex Therapeutics
SLC39A6	SGN-LIV1A	Seatle Genetics
TPBG	PF-06263507	Pfizer/Oxford Biomedica
MUC1	SAR-566658	Sanofi
MUC16	DMUC-5754A	Roche
PVRL4	AGS-22M6E	Astellas

Targeted therapies are currently unavailable for the treatment of triple-negative cancer, and patients in this group have a generally poorer prognosis [61]. Of the cell membrane targets in our list, MUC16 and the cancer/testis antigen 83 (CT83) had the most interesting profiles with high expression in triple-negative tumors and lower expression in all normal tissues examined. An ADC against MUC16 is in development by Genentech, primarily for the treatment of ovarian cancer [62]. CT83, on the other hand, is to our knowledge absent from current ADC development pipelines and has only been recently identified as a potential target in lung cancer [63]. Other genes with high differential expression ratio in triple-negative tumors versus all normal tissues included FAP, the disintegrin and metalloproteinase domain-containing protein 12 (ADAM12) and the low density lipoprotein receptor-related protein 8 (LRP8). FAP is a membrane protein of the serine protease family involved in the proteolysis of the extracellular matrix, which contributes to invasiveness in malignant cancers [64, 65]. In xenograft models, FAP5-DM1 induced long-lasting inhibition of tumor growth and complete regressions in different solid tumors with no detectable side effects [66]. LRP8 has only been recently identified as a potential target in triple-negative breast cancer [67] and is not currently, to our knowledge, considered for ADC development. ADAM12 is involved in a variety of biological processes involving cell-cell and cell-matrix interactions, and is also known as a potential drug target in breast cancer [68]. Interestingly, two of the abovementioned potential targets, namely FAP and ADAM12, are also known to be involved in the EMT [69–71].

In this study, gene expression data mining was preferred as a medium for target selection and prioritization because of the near-transcriptome coverage of modern microarray platforms and the public availability of thousands of human gene expression datasets. Mass spectrometry-based proteomics data analysis would, in contrast, provide better estimates of the quantity of interest (the actual protein abundance), although in a lower number of publically available samples, and at a typically lower resolution. Correlation between mRNA and protein levels was found to be relatively poor in a number of studies [72]. This lack of correlation may be due in part to experimental noise and biases unique to each technique. The quantification of analytes (mRNA and peptides) using indirect signals (probe and peak intensities) may also contribute to this discrepancy. In a recent study, Schwanhaüsser *et al.* [73] quantified cellular mRNA and protein expression levels and turnover in mouse cells and found that the cellular abundance of proteins was mostly controlled at the level of translation. However, Wilhelm *et al.* [74] recently analyzed mRNA and protein levels in human tissues and demonstrated that the translation rate is a constant characteristic of a transcript, and that the amount of protein in a cell is primarily controlled by transcription. This result is important: it implicates that differential expression at the mRNA level should correspond to differential abundance at the protein level, although not necessarily in a linear fashion.

Apart from tumor specificity, other factors such as tumor-specific aberrant subcellular localization may influence target selection. The glycoprotein nmb (GPNMB), for example, was characterized with high expression in all breast cancer subtypes versus normal tissues in average, but with relatively high expression in some normal tissues (breast, heart, lung and skin). GPNMB subcellular localization, however, tends to be restricted to intracellular compartments in normal cells, while being enriched on the cell surface in tumor cells [75]. In other cases, normal tissues may be considered expandables in some patients. In our list of candidate targets, some genes including, for example, the gamma-aminobutyric acid receptor subunit pi (GABRP) and cadherin 3, type 1 (CDH3), were characterized by high expression in breast cancer versus normal tissues, but with relatively high levels in normal breast. Toxicity in healthy breast tissue could be a concern and such targets may be appropriate only for subsets of patients undergoing complete mastectomy.

Exploratory gene expression analysis was performed to select candidate ADC targets, for further experimental validation at the protein level, in cell line and animal models and ultimately in clinical trials. Any of the genes in our list, EMT-related or not, respects the fundamental criterion for ADC target selection (high expression in tumor cells and lower expression in normal tissues). Although membrane proteins represent more attractive targets for use in ADC internalization strategies, extracellular proteins may also prove useful, given that strategies such as that reported in [35] are developed and tested. Some of the selected ADC targets would be interesting candidates in triple-negative tumors. These deserve even higher attention for further experimental testing and validation, because triple-negative breast cancer patients have currently no targeted therapy options, and have a generally poorer prognosis. The same approach could be used to mine gene expression data in other cancers, and to identify additional targets for ADC development.

## MATERIALS AND METHODS

### Microarray data collection

Raw microarray data corresponding to 101,334 samples analyzed using the Affymetrix Human Genome U133 Plus 2.0 platform [16] was obtained from the GEO database [15] using custom Perl scripts. Additional, tumor-derived cell line gene expression profiling data was downloaded from the CellMiner database [76] and the caBIG database [77]. To have a complete set with three replicates for each of the 359 unique cell lines, additional samples from GEO (GSM274690, GSM274785, GSM559851, GSM886956, GSM887076, GSM887415, GSM887651) were added to those datasets. Data analysis was performed using R version 3.1.1 [78] and Bioconductor version 3.0 [79]. CEL files were read in R with the affy package [80] using BrainArray Entrez Gene custom chip definition file version 18 [47], and normalized using the MAS 5.0 algorithm [81]. Pre-processed microarray data was stored in indexed binary files for efficient storage and retrieval.

### Metadata analysis

Metadata associated with 101,334 samples (sample id, series id, title, description, source, characteristics) and the corresponding 3,643 series (series id, title, summary, overall design, pubmed ids) were retrieved using GEOmetadb [18] and stored in a SQLite database. Publications (pubmed id, year, journal, title, abstract) linked with the experiments were retrieved using Bioperl utilities [82] and stored in the database. A new table was created for the purpose of sample re-annotation. This table was populated using dedicated Java software comprising a search engine translating Boolean queries into SQL statements and a spreadsheet-like interface allowing direct and programmatic editing of annotations. Breast cancer samples (4,853 tumor) and normal tissue samples (1,067 blood, 291 bone marrow, 526 breast, 353 colon, 50 heart, 279 kidney, 287 liver, 478 lung, 85 pancreas, 334 skin and 25 stomach) were selected from over 200 experiments using this tool (Supplementary Data, GEO Series).

### Breast sample classification

Gene expression data was collected for a total of 5,379 breast samples. Receptor status data (ER, PR and HER2) was available in sample annotations for 3,500 samples (1,766 complete, 1,734 partial). For each receptor, two locally adaptive kernel densities were estimated in annotated samples (positive, negative), and receptor status in other samples was predicted by assigning the label corresponding to the maximum posterior [83]. Samples were then assigned to one of four classes: normal (annotated as normal), luminal (ER+ and/or PR+), HER2+ (ER-, PR-, and HER2+) and triple-negative (ER-, PR-, HER2-). Classes were compared one-against-one (OAO) and one-against-all (OAA) for multiclass classification [84]. Filter-based feature selection was performed by selecting the top ${\left(2^{k}\right)}_{k=1}^{10}$ features ranked using three different statistics: *q*-values derived from linear models for microarray (Limma) moderated *t*-test [24, 85], the overlapping coefficient of locally adaptive kernel density estimates [30, 31], and the weight of support vector machines (SVM) [29]. Locally adaptive kernel densities and overlapping coefficients were computed using an in-house R package implemented with Rcpp [86]. The weight of SVM were computed using the e1071 R package [87]. To estimate classification error for combinations of feature selection and classification algorithms, repeated (ten times) five-fold stratified cross-validation was performed [88, 89]. Classification was achieved using six algorithms implemented in the RWeka package [90]: bagging [91], J48 (C4.5 decision tree) [92], *k*-nearest neighbors (KNN) [93], naive Bayes [94], random forests (RFO) [95] and support vectors machines (SVM) [96]. For the OAO classification, class labels were assigned where the maximum label allocation was reached (three in six binary classification problems). For OAA classification, class labels were assigned where only one label was assigned among four classification problems. For each combination of factors in the cross-validation, the accuracy was calculated as the sum of correct predictions divided by the total number of predictions. The performance of combinations of feature selection methods, classification algorithms, number of features and classification strategies was compared using analysis of variance, and the best performing combinations were retained for ensemble classification [97]. An iterative method [98] was used to assign labels to breast samples. Labels assigned with high confidence (>95% of votes) by an ensemble of experts (36 votes from the combination of three feature selection methods, two classification methods, and six increasing number of features) were fed back into the data and used for subsequent feature selection and training of the classifiers. This procedure was repeated until the number of predictions was stable over a number of iterations, or until complete convergence was achieved.

### Cell line classification

Results from Ross *et al.* [46] were used to label NCI60 cell lines according to patterns of expression in the epithelial, mesenchymal, melanoma and leukemia gene clusters (Supplementary, Table S1). Cell lines having high levels of expression of genes in the epithelial cluster and low expression in the mesenchymal cluster were labeled as epithelial, and cell lines characterized by the opposite pattern were labeled as mesenchymal. Cell lines having intermediate expression profiles between these two classes were labeled as mixed. Cell lines having levels of expression characteristic of the melanoma and leukemia gene clusters were labeled as melanoma and leukemia, respectively. Assigned class labels were validated using repeated cross-validation as described above, with the difference that feature selection was performed separately in three sets of replicates, and validation was performed in the two remaining sets. For the OAO classification, class labels were assigned where the maximum label allocation was reached (four in ten binary classification problems), and for the OAA classification, class labels were assigned where only one label was assigned among five classification problems. For the classification of unlabeled cell lines, starting with the NCI60 panel, each cell line replicate was randomly assigned to one of three sets, and for each set, filter-based feature selection was performed using three statistics as described above. Classifiers were trained on each set of labeled replicates, and predictions were made on each unlabeled cell line replicate. Iterative ensemble classification method was used as described above to assign labels to the new cell lines using 324 votes from an ensemble of experts (combination of three sets of labeled replicates, three feature selection methods, two classification methods, six increasing number of features, and three unlabeled replicates).

### Target selection and prioritization

To identify genes specific to or overexpressed in breast cancer, expression profiles in each molecular subtype were compared with gene expression in major organs and tissues were toxicity would likely be a serious concern (blood, bone marrow, colon, heart, kidney, liver, lung, pancreas, skin and stomach). An initial filtering was done to retrieve genes with a maximum ratio (normal/cancer) of 2, an average ratio (cancer/normal) greater than 2 and an average overlapping coefficient [30, 31] smaller than 0.6. Subcellular localization data was obtained from Uniprot annotations [50] and the Gene Ontology Annotation (GOA) database [49]. For each molecular subtype, protein-coding genes were ranked according to the mean rank of ratios in cancer versus normal tissues, and the top 50 (balanced with respect to the three subtypes) cell membrane or extracellular protein-coding genes were retained. Batch effects were evaluated by extracting the first two principal components (52% of total variance) from the 1,000 genes with highest total variance in breast cancer and normal tissues, and visualizing patterns associated with tissue source, sample status and experiments. In addition, batch effects were quantified using linear mixed models [99] with tissue and status as fixed effects and series as random effect, and were compared with the amplitude of differential expression in the breast cancer subtype(s) in which the target was identified versus normal tissues. To prioritize targets linked with EMT, differential gene expression analysis was performed between epithelial and mesenchymal tumor-derived cell lines. The same parameters (expression ratio > 2 and overlapping coefficient < 0.6) were used for the selection of EMT-related targets.

## SUPPLEMENTARY MATERIAL AND METHODS, TABLES AND FIGURES


