# Supplementary material for: Computational selection of antibody-drug conjugate targets for breast cancer
Source: Oncotarget. 2015 Dec 19;7(3):2555–71. doi: 10.18632/oncotarget.6679 (PMC4823055; doi:10.18632/oncotarget.6679)
Supplement: Supplementary file 1 [file oncotarget-07-2555-s001.pdf]

SUPPLEMENTARY MATERIAL AND METHODS, TABLES AND FIGURES

GEO series.

GSE10006, GSE10041, GSE10191, GSE10281, GSE10616, GSE10714, GSE10715, GSE10780, GSE10791, GSE10799, GSE10810, GSE11001, GSE11024, GSE11045, GSE11151, GSE11375, GSE1145, GSE11504, GSE11784, GSE11831, GSE12187, GSE12276, GSE12763, GSE12790, GSE12917, GSE13159, GSE13204, GSE13355, GSE13367, GSE13501, GSE13787, GSE13849, GSE13904, GSE13933, GSE14334, GSE14580, GSE14668, GSE14762, GSE14905, GSE15061, GSE15471, GSE15543, GSE15605, GSE15932, GSE16161, GSE16391, GSE16446, GSE16515, GSE16538, GSE16728, GSE16879, GSE17183, GSE17312, GSE17476, GSE17700, GSE17816, GSE17818, GSE17895, GSE17907, GSE18105, GSE18123, GSE18728, GSE18781, GSE18842, GSE18864, GSE18995, GSE19188, GSE19249, GSE19615, GSE19650, GSE19697, GSE19743, GSE19804, GSE20307, GSE20489, GSE20685, GSE20711, GSE20713, GSE20916, GSE2109, GSE21422, GSE21510, GSE21610, GSE21653, GSE21816, GSE22035, GSE22229, GSE22459, GSE22513, GSE22544, GSE22598, GSE22619, GSE22780, GSE22840, GSE23177, GSE23343, GSE23593, GSE23604, GSE23630, GSE23720, GSE24006, GSE24206, GSE24223, GSE26049, GSE26378, GSE26440, GSE26457, GSE26554, GSE26639, GSE26713, GSE26910, GSE28536, GSE28583, GSE28619, GSE28694, GSE28750, GSE28796, GSE28821, GSE28826, GSE28844, GSE29044, GSE29431, GSE29691, GSE29721, GSE29722, GSE29819, GSE29919, GSE30010, GSE30418, GSE31192, GSE31210, GSE31448, GSE32646, GSE32676, GSE32688, GSE32887, GSE32924, GSE33075, GSE33356, GSE33532, GSE33658, GSE34748, GSE3526, GSE35925, GSE36076, GSE36771, GSE36774, GSE37031, GSE37267, GSE37364, GSE3744, GSE37455, GSE37460, GSE37463, GSE37768, GSE38554, GSE38713, GSE38941, GSE39612, GSE40231, GSE40791, GSE40837, GSE4107, GSE41328, GSE4183, GSE42109, GSE42114, GSE42568, GSE43346, GSE43365, GSE43502, GSE4488, GSE45267, GSE45436, GSE46474, GSE47109, GSE47389, GSE48060, GSE48311, GSE48390, GSE48391, GSE50567, GSE50628, GSE50948, GSE51024, GSE52322, GSE52360, GSE52746, GSE53223, GSE5350, GSE53757, GSE5460, GSE54837, GSE55201, GSE55594, GSE56808, GSE5764, GSE58294, GSE58792, GSE59312, GSE6351, GSE6460, GSE6532, GSE6764, GSE7023, GSE7307, GSE7515, GSE7869, GSE7904, GSE8050, GSE8121, GSE8545, GSE8581, GSE8671, GSE8977, GSE9086, GSE9195, GSE9196, GSE9254, GSE9452, GSE9489, GSE9493, GSE9686, GSE9692.

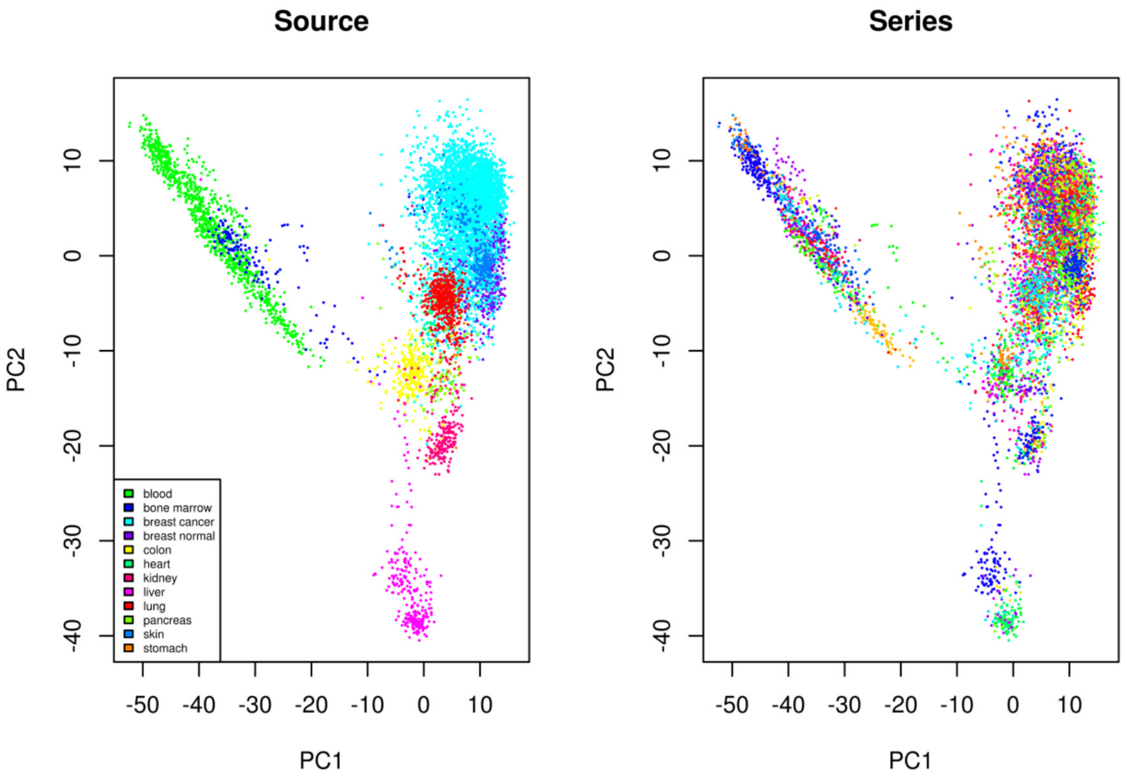

Supplementary Figure S1: Plot of the first and the second principal components in 8,628 breast cancer and normal tissue samples. Left, samples are colored by organ/tissue source. Right, samples are colored by series (experiments).

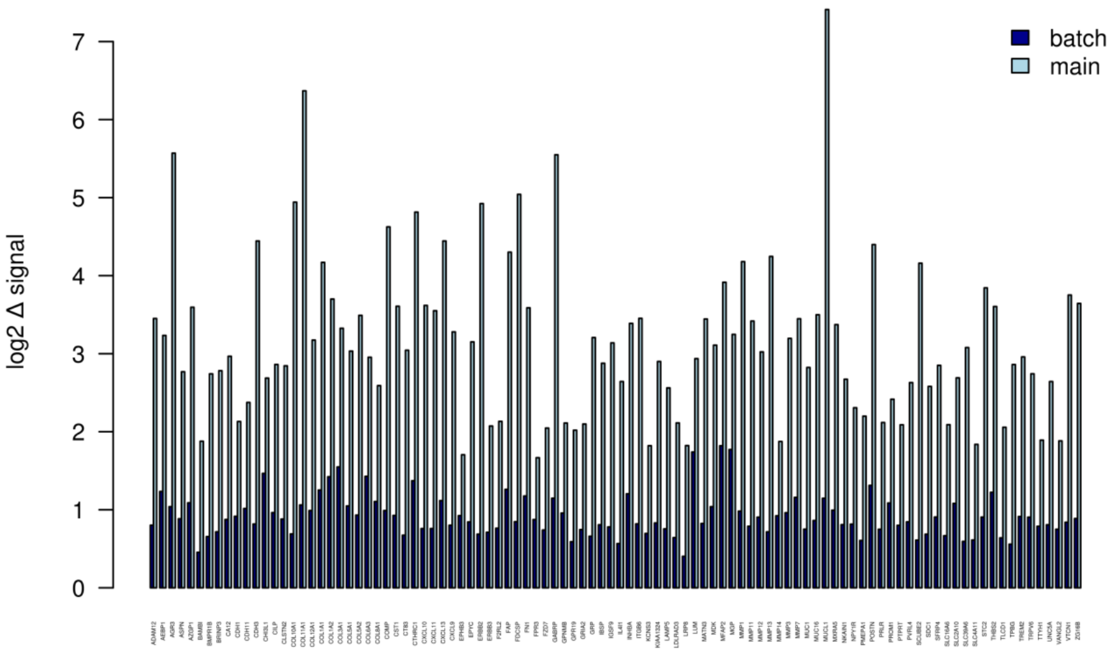

Supplementary Figure S2: Barplot of experiment (batch) effect compared to the amplitude of differential expression in cancer versus normal tissues samples (main) for 100 candidate targets.

Supplementary Table S1: Assigned NCI60 cell line classes.

Class	Cell lines
Epithelial	COLO205, HCC2998, HCT116, HCT15, HT29, KM12, MCF7, NCIH322M, OVCAR3, OVCAR4, SW620, T47D
Mesenchymal	7860, A498, ACHN, BT549, CAKI1, HOP62, HOP92, HS578T, LOXIMVI, MDAMB231, NCIADRRES, NCIH226, OVCAR8, RXF393, SF268, SF295, SF539, SN12C, SNB19, SNB75, TK10, U251, UO31
Mixed	A549, DU145, EKVX, IGROV1, NCIH23, NCIH460, NCIH522, OVCAR5, PC3, SKOV3
Leukemia	CCRFCEM, HL60, K562, MOLT4, RPMI8226, SR
Melanoma	M14, MALME3M, MDAMB435, SKMEL2, SKMEL28, SKMEL5, UACC257, UACC62

Supplementary Table S2: Predicted cell line classes in the GlaxoSmithKline collection.

Class	Cell lines
Epithelial	22RV1, 5637, 647V, AGS, ASPC1, BEWO, BFTC905, BT20, BT474, C4I, C4II, CAHPV10, CAOV3, CAPAN1, CAPAN2, CHAGOK1, COLO201, COLO668, CORL105, CORL23, DETROIT562, DMS153, DMS53, DOTC2, DU4475, EFM19, FADU, HCC1143, HCC1599, HCC1937, HCC1954, HCC2157, HCC2218, HCC38, HCT8, HEC1A, HPAC, HPAFII, HT1197, HT1376, HT3, HUPT4, JAR, JEG3, KATOIII, KPL1, KYSE30, LS1034, LS174T, MDAMB175VII, MDAMB453, MT3, NCIH1395, NCIH1437, NCIH1573, NCIH1623, NCIH1650, NCIH1666, NCIH1838, NCIH1993, NCIH2009, NCIH2087, NCIH2122, NCIH2126, NCIH2170, NCIH2347, NCIH322, NCIH358, NCIH441, NCIH508, NCIH630, NCIH747, OE19, OE21, OE33, RL952, RWPE1, SCABER, SKMEL3, SNU16, SW1116, SW1417, SW1463, SW1990, SW403, SW48, SW780, SW837, SW900, SW948, SW954, T84, UACC812, UACC893, WIDR, YPAC
Mesenchymal	639V, 769P, A172, BHT101, CAKI2, CAL54, CAL62, CALU1, CCFSTTG1, CGTHW1, DBTRG05MG, DKMG, ES2, GCT, H4, HCC1395, HDMYZ, HOS, HT1080, J82, KHOS240S, MG63, NCIH1792, NCIH2052, NCIH2228, RD, SJSA1, SKLMS1, SKMES1, SNU182, SNU387, SNU423, SNU449, SNU475, SW1088, SW1353, SW1783, SW684, SW756, SW872, SW982, UMUC3, WI38
Mixed	A204, A427, A673, A7, AN3CA, BE2C, BM1604, C33A, C3A, CALU6, CHL1, CHP212, COLO320DM, COLO320HSR, COLO704, CORL279, D283MED, D341MED, DMS114, DMS273, DMS79, G401, G402, HEC1B, HEP3B, HEPG2, HMCB, MCIXC, MESSA, MIAPACA2, NCIH1092, NCIH1155, NCIH1355, NCIH1436, NCIH1581, NCIH1618, NCIH1651, NCIH1694, NCIH1703, NCIH1770, NCIH1793, NCIH187, NCIH1930, NCIH2030, NCIH2081, NCIH2107, NCIH2171, NCIH2195, NCIH2405, NCIH295R, NCIH446, NCIH524, NCIH650, NCIH661, NCIH69, NCIH716, NCIH720, NCIH748, NCIH810, NCIH82, NCIH838, OV90, RDES, RKOE6, SHP77, SIHA, SJRH30, SKNAS, SKNDZ, SKNEP1, SKNFI, SKUT1, SNU1, SNU398, SNU5, SW1573, TT, Y79
Leukemia	1A2, ARH77, BC1, BC2, BC3, BDCM, BV173, CA46, CEMC1, CESS, CMLT1, CROAP2, CROAP5, DAUDI, DB, DG75, DOHH2, EB1, EB2, EB3, EM2, GA10, GDM1, HEL9217, HH, HSSULTAN, HT, HUNS1, HUT78, JIYOYE, JM1, JRT3T35, JVM3, KASUMI2, KG1, KU812, L428, MC116, MCCAR, MEC1, MEG01, MHHPREB1, MJ, ML2, MOLT16, MV4II, NALM1, NALM6, NAMALWA, NC37, P3HR1, PLB985, RAJI, RCHACV, REC1, RL, RPMI6666, SEM, SKO007, ST486, SUDHL10, SUDHL16, SUDHL5, SUDHL6, TANOUE, THP1, TOLEDO, U266B1
Melanoma	A101D, A375, C32TG, COLO829, SH4, SKMEL1, WM115
